# miRNAs: From Master Regulators of Gene Expression to Biomarkers Involved in Intercellular Communication

**DOI:** 10.3390/biomedicines12040721

**Published:** 2024-03-25

**Authors:** Elena Levantini, Milena Rizzo

**Affiliations:** 1 Institute of Biomedical Technologies, CNR, Via Moruzzi 1, 56124 Pisa, Italy; elena.levantini@itb.cnr.it; 2 Institute of Clinical Physiology, CNR, Via Moruzzi 1, 56124 Pisa, Italy

## 1. Introduction

MicroRNAs (miRNAs) are non-coding RNAs that act as master regulators of gene expression, fine-tuning the activity of thousands of genes in our cells, by modulating gene expression at the post-transcriptional level. They play a fundamental role in the regulation of almost all physiological processes, and their alteration can contribute to the development of several diseases.

This Special Issue collects some of the latest advances in the field, focusing on three main aspects: (i) the involvement of miRNAs in human disease, (ii) the role of extracellular miRNAs (ex-miRNAs) in intercellular communication and (iii) the use of ex-miRNAs as biomarkers to diagnose and monitor diseases in different body fluids. Moreover, this Special Issue also covers some of the “less conventional” aspects of miRNA research, such as the discovery of both small nucleolar (sno)-derived RNAs and transposable elements as novel players in the miRNA regulatory network. These findings reveal the diversity and complexity of the miRNA regulatory network, which is constantly evolving and expanding.

## 2. Extracellular miRNAs

In 2008, it was discovered that miRNAs are also present in the extracellular space (ex-miRNAs), creating a new dimension of gene regulation that crosses the boundaries of cells and tissues, thus opening up a new field of research with great potential for biomedical applications. Ex-miRNAs are released from cells and circulate in various biological fluids (blood, saliva, urine, milk, cerebrospinal fluid, etc.). For this reason, they are also called circulating miRNAs (c-miRNAs). They carry information about our health status and can be used as biomarkers for different diseases and disorders, thanks to their high stability and abundance in these fluids. Furthermore, the analysis of c-miRNAs has the clear advantage of being minimally invasive compared to tissue biopsy (e.g., [1,2]).

In this Special Issue, several papers explore the potential of c-miRNAs as biomarkers in different scenarios, such as pregnancy, brain damage, ovarian cancer, and breast milk. For instance, Thibeault et al. [3] identified some plasma miRNAs associated with the maternal body mass index in the first trimester of pregnancy, many of which were related to fatty acid and lipid metabolism according to an in silico analysis. Robles et al. [4] identified a set of c-miRNAs whose level changed in the serum of a mouse model of intracerebral hemorrhage (ICH), which could be evaluated as potential biomarkers of brain injury. Robotti et al. [5] reviewed how salivary miRNAs can act as potential biomarkers of ovarian cancer, emphasizing why saliva is a reliable and easy-to-manage source of biomarkers in tumor diagnosis. Kondracka et al. [6] reviewed the recent findings on human breast milk miRNAs. The authors highlighted their possible role in shaping the development of the infant’s immune systems and how they are associated with some diseases in both infants and mothers, including breast neoplasms and neonatal jaundice. Related to this aspect, Pomar et al. [7] showed that changing the diet of diet-induced obese mice during lactation can reduce the abnormal level of miRNAs in their mammary glands, but not in their milk.

However, despite more than a decade of research, using c-miRNAs as biomarkers is not an easy task. There are still technical hurdles to overcome, such as the accuracy and reproducibility of their quantification [8,9]. Luckily, some technical advances have been made, especially with digital PCR (dPCR), which offers high sensitivity and specificity in detecting low-level miRNAs in human plasma. D’Alessandra et al. [10] confirmed the accuracy of dPCR in this context and also proposed a faster and simpler way to quantify circulating miRNAs with dPCR, without the need to extract them first.

Another aspect of ex-miRNAs, which is still debated, is their role in intercellular communication, either as free molecules or as encapsulated vesicles (such as exosomes or other types of extracellular vesicles). There are many indications that ex-miRNAs can act as signaling molecules in both an autocrine/paracrine and an endocrine manner, influencing physiological and pathological processes (e.g., [11,12,13]), including drug resistance (e.g., [14]). Zeng et al. [15] reviewed some examples of exosomal miRNAs that play a role in cell–cell communication in pathological states, highlighting their therapeutic potential. The authors focused on miRNAs secreted by mesenchymal stem cells and their potential application in bone regeneration and various diseases (such as cancer, Alzheimer’s disease, spinal cord injury, ischemia) treatment. Barbosa et al. [16] showed that the secretome isolated from miR-124-silenced amyotrophic lateral sclerosis (ALS) motoneurons, if injected into the spine of ALS mice (at the early stage), was able to hamper the progression of the disease, improving locomotor behaviors and preventing motoneuronal dysfunction by reducing neurodegeneration.

## 3. miRNAs in Human Diseases

Many pieces of evidence support the hypothesis that miRNAs are deregulated in all hallmarks of cancers. In this Special Issue, you can find some papers that show how miRNAs can act as both villains and heroes in different types of cancer. For example, Panella et al. [17] demonstrated how miR-22 can make triple-negative breast cancer cells more aggressive by promoting epithelial–mesenchymal transition (EMT). The authors also demonstrated how pharmacologically inhibiting miR-22 improved the survival of mice with breast tumors, highlighting the possible use of miRNAs as targets for therapeutic development [18]. In their review, Nguyen et al. [19] summarized how miR-29s can have opposite roles in different human cancers, acting as either oncogene or tumor suppressors. Chiantore et al. [20] defined a miRNA signature in actinic keratosis, a skin condition that can lead to cancer, that may affect the pathways involved in tumor development. De Almeida et al. [21] reported the most relevant findings on the epigenetic features of uterine leiomyosarcomas and endometrial stromal sarcoma, two rare and aggressive cancers. Among them, several miRNAs were found to be altered in these tumors. Finally, Galardi et al. [22] reviewed the most recent data on the role of circular RNAs (circRNAs) in pediatric cancer. CircRNAs are long (>200 bp) non-coding RNAs that regulate gene expression at various levels (by affecting the transcription efficiency, by interacting with proteins and modifying their activity, etc.) [23]. One of the most commonly reported functions of circRNAs, even for pediatric cancer, is trapping miRNAs (miRNA sponging). Recently, this function has been questioned, and the most likely conclusion is that for circRNAs to have a significant biological effect, they should either contain many miRNA binding sites or be highly expressed [23].

miRNAs are also involved in many other diseases and disorders. In this Special Issue, some papers show how miRNAs can affect the development and function in different organs and systems. For instance, Leavy et al. [24] reported that the impairment of normal brain maturation caused by early life brain injury (such as hypoxia) is accompanied by modification of miRNA expression, possibly driven by cMYC. In their review, Piquer-Gil et al. [25] focused on the importance of the balance between two key pathways (Wnt/β-catenin and Hippo pathways) in the progression of the pathological arrhythmogenic cardiomyopathy (ACM) phenotype, a form of heart disease that can cause sudden death. The authors, by using data from cancer research, hypothesized that non-coding RNA (including miRNAs) could play a role in regulating these pathways in ACM. Finally, An et al. [26] investigated the association of specific miRNA polymorphisms with pregnancy loss.

## 4. “Non-Canonical” miRNAs and miRNA Regulation

In this Special Issue, authors discuss i) a neglected class of non-coding RNA with miRNA-like functions and ii) a new function of transposons in miRNA sponging.

The review by Coley et al. [27] focused on sno-derived RNAs (sdRNAs), small RNAs that originate from small nuclear RNAs (snoRNAs), which can act as miRNA-like molecules. This review summarized the recent literature on the role of sdRNAs in cancer gene regulation, including miRNAs derived from snoRNA transcripts. The authors highlighted the relevant role of this small non-coding RNA category, especially in cancer pathology, and urged the boosting of research in this novel field.

The paper by Esposito et al. [28] studied a possible new function of transposons (TEs), which are mobile genetic elements that can move around the genome and sometimes affect the expression of nearby genes. TEs, depending on where they jump, can insert functional domains such as miRNA binding sites transforming transcripts in miRNA sponges (see reference in the paper). This is part of a network of competitive endogenous RNAs (ceRNAs), where different RNA molecules compete for the same miRNAs and influence their availability and activity [29]. The authors investigated the role of TEs as miRNA sponges by analyzing in silico transcriptomic data of different cellular conditions in which a specific type of TE, called LINE L1, was more active. LINE L1, the most abundant and active TE in humans, can copy and paste itself to new locations in the genome. The authors found that, in a cellular condition where LINE L1 was overactive, genes that had many miRNA binding sites in common with LINE L1 were more upregulated compared to genes with fewer shared miRNA binding sites. This suggests that an increase in LINE L1 could increase specific miRNAs’ sequestration, derepressing their targets. The authors proposed that this could be a new miRNA regulation modality that could impact both health and disease. However, they also acknowledged that this phenomenon needs to be experimentally validated and further investigated.

## 5. Conclusions

In conclusion, this Special Issue covers the current state of the art on miRNA research, providing novel insights on miRNA regulation and function that could advance our understanding and applications of these versatile biomolecules. We hope that this collection of papers will inspire further research and innovation in this exciting and promising field.
